# N-Glycosylation of the alpha subunit does not influence trafficking or functional activity of the human organic solute transporter alpha/beta

**DOI:** 10.1186/1471-2121-9-57

**Published:** 2008-10-10

**Authors:** Carol J Soroka, Shuhua Xu, Albert Mennone, Ping Lam, James L Boyer

**Affiliations:** 1 Department of Medicine, Yale University School of Medicine, New Haven, CT, USA

## Abstract

**Background:**

The organic solute transporter (OSTα-OSTβ) is a heteromeric transporter that is expressed on the basolateral membrane of epithelium in intestine, kidney, liver, testis and adrenal gland and facilitates efflux of bile acids and other steroid solutes. Both subunits are required for plasma membrane localization of the functional transporter but it is unclear how and where the subunits interact and whether glycosylation is required for functional activity. We sought to examine these questions for the human OSTα-OSTβ transporter using the human hepatoma cell line, HepG2, and COS7 cells transfected with constructs of human OSTα-FLAG and OSTβ-Myc.

**Results:**

Tunicamycin treatment demonstrated that human OSTα is glycosylated. In COS7 cells Western blotting identified the unglycosylated form (~31 kD), the core precursor form (~35 kD), and the mature, complex glycoprotein (~40 kD). Immunofluorescence of both cells indicated that, in the presence of OSTβ, the alpha subunit could still be expressed on the plasma membrane after tunicamycin treatment. Furthermore, the functional uptake of ^3^H-estrone sulfate was unchanged in the absence of N-glycosylation. Co-immunoprecipitation indicates that the immature form of OSTα interact with OSTβ. However, immunoprecipitation of OSTβ using an anti-Myc antibody did not co-precipitate the mature, complex glycosylated form of OSTα, suggesting that the primary interaction occurs early in the biosynthetic pathway and may be transient.

**Conclusion:**

In conclusion, human OSTα is a glycoprotein that requires interaction with OSTβ to reach the plasma membrane. However, glycosylation of OSTα is not necessary for interaction with the beta subunit or for membrane localization or function of the heteromeric transporter.

## Background

The organic solute transporter (OSTα-OSTβ) is a heteromeric transporter of bile acids and other organic solutes and steroids. In the human, OSTα-OSTβ is found predominantly in epithelial cells of liver, intestine, kidney, adrenal gland and testis[1]. It is expressed on the basolateral membrane of these cells and has been shown to transport estrone 3-sulfate, digoxin, dehydroepiandrosterone 3-sulfate, prostaglandin E_2 _and a variety of bile acids [1-3]. Regulation of this basolateral transporter is through the action of the bile acid-activated nuclear receptor, the farnesoid × receptor (FXR) [4]. Thus, conditions of cholestasis have been shown to result in up-regulation of OSTα-OSTβ at both the mRNA and protein levels [4]. Recently the importance of this transporter in intestinal bile acid transport and in the enterohepatic circulation has been confirmed in Ostα-/- mice [5]. Data from studies of these mice highlight the role of Ostα-OSTβ and FGF15 in regulating hepatic bile acid synthesis.

It was noted early on that transport activity required the coexpression of two distinct gene products. The first, Ostα, is a predicted 340-amino acid protein with seven membrane spanning domains and the second, Ostβ, is a 128-amino acid, single membrane spanning protein [2]. Transport is bidirectional across the plasma membrane, and most likely occurs by facilitated diffusion of substrates down their electrochemical gradients [3]. Plasma membrane localization and functional activity requires the expression of both subunits [3,6-8]. Several groups have shown that the functional requirement for co-expression of both subunits is associated with the physical association of the two proteins [7,8]. Dawson and colleagues demonstrated that mouse Ostβ was necessary for mouse Ostα to acquire N-glycosylation in transfected HEK293 cells, thus suggesting that the beta subunit is acting as a chaperone to allow the alpha subunit to exit the ER [6]. Quality control at the level of the ER can involve many different mechanisms. Newly synthesized proteins must be folded correctly and, in some cases, must be assembled into multimeric protein complexes in order to be trafficked to the Golgi and plasma membrane. If this does not occur the protein may be ubiquitinated and designated for degradation. Thus, the chaperone activity of OSTβ may require a properly folded alpha subunit or may aid in the folding of the peptide. Alternatively, the protein-protein interaction may mask a retention/retrieval motif or reveal a forward trafficking motif in the alpha subunit. Recent work shows that both subunits must be expressed in order to prevent degradation of the other subunit, suggesting a specific interaction between the two proteins [5,7]. Sun and colleagues have suggested that OSTβ is interacting with the N-terminus, and not the C-terminus, of OSTα [8]. This raises the question of whether the glycosylation of the alpha subunit could influence the interaction with the beta subunit and, thus, affect membrane localization and function of the intact transporter. Therefore, in this study we have sought to examine more fully the interaction of OSTα and OSTβ in two mammalian expression systems where we can look at both the endogenous and exogenous, transfected expression of the human transporter.

## Methods

### Cell Culture

The human hepatocellular carcinoma cell line, HepG2, and the monkey kidney cell line, COS7, were acquired from ATCC (Manassas, VA). HepG2 cells were cultured in MEM with non-essential amino acids (ATCC) containing 10% FBS and 1% penicillin-streptomycin, at 37°C with 5% CO_2_. COS7 cells were cultured in DMEM containing 10% FBS and 1% penicillin-streptomycin, at 37°C with 5% CO_2_.

### Cell treatment

After HepG2 cells reached ~70% confluence, they were washed and cultured in fresh medium containing 10% charcoal-stripped serum in the presence or absence of 50 μM chenodeoxycholate (CDCA) (Sigma, St Louis, MO), or 2 μM 6-ethyl CDCA (Dr. Roberto Pellicciari, Universita Di Perugia, Italy). Twenty four to forty-eight hours after addition of CDCA, RNA and protein were isolated or cells were fixed for immunofluorescence as described below. To inhibit glycosylation, tunicamycin (Sigma) was added at concentrations indicated in the figure legends 6 hrs after the addition of CDCA treatment in HepG2 cells or 4 hrs after the initiation of transfection in COS7 cells.

Lysates from COS7 cells transfected for 48 hrs with OSTα-FLAG and OSTβ-MYC were digested with peptide:N-glycosidase F (PNGase F) and endoglycosidase H (EndoH) according to the manufacturer's instructions (New England Biolabs) and subjected to SDS-PAGE as described below.

### Cloning human OST alpha, beta and vector constructs

HepG2 cell cDNA was used as a template. We generated a 1.03 kb cDNA fragment encoding the full-length of human OSTα and a 0.4 kb cDNA fragment encoding the full-length of human OSTβ by PCR. The primers for amplification of human OSTα and OSTβ were based on the published human sequences (GenBank accession number AK172837 and AY194242). The forward primer OSTα 5'-GCTTGGTACCATGGAGCCGGGCAGGACCCAGATAA-3' and the reverse primer OSTα 5'-CCGCTCGAGTTACTTGTCATCGTCGTCCTTGTAATCCCCGGCTTTGAGGTTCAAGTCCAGGTC-3' were used. The forward primer OSTβ 5'-GCTGGATCCACCATGGAGCACAGTGAGGGGGCTCC-3' and the reverse primer OSTβ 5'-GCACTCGAGGCTCTC AGTTTCTGGTACATCCGG-3' were used. The amplified cDNA fragment encoding the full-length of OSTα was then subcloned into the Kpn I and Xho I sites of the mammalian expression vector pcDNA3.1 (Invitrogen) and the cDNA fragment encoding the full-length of OSTβ was subcloned into the BamH I and Xho I sites of pcDNA3.1 Myc/His vector (Invitrogen). PcDNA3.1-OSTα-FLAG and pcDNA3.1-OSTβ-Myc/His were sequenced using Yale Keck DNA sequencing facility. The coding sequences were identical to the published sequences with the GenBank accession numbers for OSTα [AY194243] and for OSTβ [BC103842].

COS7 cells were transfected with FuGene 6 (Roche) using 1 μg OSTα-FLAG or OSTβ-Myc DNA/9 cm^2 ^surface area, according to manufacturer's instructions. pcDNA vector control (1 μg DNA) was used when only one subunit was transfected. Cells were harvested 24-48 hr after transfection, as described for Western blotting or immunofluorescence.

### Quantitative RT-PCR

Cells were extracted with Trizol (Invitrogen, Carlsbad, CA) and RNA was isolated according to manufacturer's instructions. Quantitative RT-PCR was carried out as described previously [4] using Applied Biosystems 7500 DNA sequence detector system with TaqMan universal master mix (Applied Biosystems, Foster City, CA). Specific primer pairs for hOSTα and hOSTβ were the same as previously described [4].

### Western blot/Immunoprecipitation

Cells were washed with PBS and then extracted directly in RIPA buffer (25 mM Tris, pH 7.2, 150 mM NaCl, 10 mM EDTA, 1% Triton X-100, 1% deoxycholate, 0.1% SDS) or in 1% Triton X-100, 50 mM Tris HCl, pH 7.4,150 mM NaCl, 1 mM EDTA for immunoprecipitation. Lysates were centrifuged at 10,000 × g for 20 min and the supernatant was collected for analysis using SDS-PAGE. Immunoprecipatation was performed using anti-FLAG affinity gel (M2, Sigma, St Louis, MO) or anti-Myc polyclonal antibody (abcam, Cambridge, MA) and Protein A/G beads (Santa Cruz). Lysates were precleared and negative controls were performed with non-specific anti-mouse IgG. In the case of immunoprecipitation of endogenous protein from HepG2 cells, a Native IgG kit from Pierce was used with polyclonal antibodies raised against OSTα (hOSTα-327) and OSTβ (hOSTβ-1) provided by Ned Ballatori (Rochester, NY).

Pulse-chase experiments were carried out in COS7 cells 24 hr after transfection. After incubation for 15 min with Cys/Met minus media, cells were pulsed for 15 min with media containing 135 μCi ^35^S Trans-label (MP Biomedicals, Solon, OH). Cells were either extracted immediately in 1% Triton X-100, 50 mM Tris Hcl, pH 7.4,150 mM NaCl, 1 mM EDTA or chased for 2 hr in complete media. Immunoprecipitation was carried out as described above.

### Immunofluorescence

Cells were fixed with cold methanol for 10 min or with 4% paraformaldehyde for 15 min. Quenching of non-specific fluorescence in formaldehyde fixed cells was done with 50 mM NH_4_Cl for 20 min prior to blocking 20 minutes in blocking buffer (PBS, 1% BSA, 0.05% Triton X-100). In the case of OSTβ, non-permeablized conditions using no detergent was found to give better surface labeling. Primary antibody was diluted in blocking buffer and incubated on the cells for 2 hours at room temperature. After washing in PBS, secondary antibody (Alexa 594 or 488 anti-IgG (Invitrogen)) was incubated for 1 hour at room temperature. Fluorescence was visualized with a Zeiss LSM510 (Carl Zeiss Inc, Thornwood, NY) confocal microscope and images processed with Photoshop (Adobe, Mountainview, CA).

### Transport assay

HepG2 cells were cultured in 35 mm dishes as described above. At ~70% confluency 50 μM CDCA or vehicle was added and the culture continued for 48 hrs. ^3^H-Taurocholate (1 μM) or ^3^H-estrone 3-sulfate (15 nM) were made up in transport buffer (116 mM NaCl, 5.3 mM KCl, 1.1 mM KH2PO4, 0.8 MgSO4.7H2O, 1.8 mM CaCl, 11 mM glucose, 10 mM HEPES) and warmed to 37°C. For each time point, triplicate dishes were washed 3 times with warm transport buffer alone and then incubated for the given time with 1 ml transport buffer containing ^3^H-substrate. Uptake of substrates was stopped by rapid addition and aspiration of 1 ml of cold transport buffer three times. Cells were lysed with 1 ml 0.5% Triton X100. Cell lysates (600 μl) were combined with OptiFluor scintillation fluid (5 ml) and counted in a PerkinElmer WinSpectral LSC (PerkinElmer, Waltham, MA). Protein content of the lysates was determined with the BCA reagent (Pierce Biotechnology, Rockford, IL) and used to normalize the counts.

## Results

### N-glycosylation of OSTα is not required for plasma membrane expression in HepG2 cells

**Figure 1:**
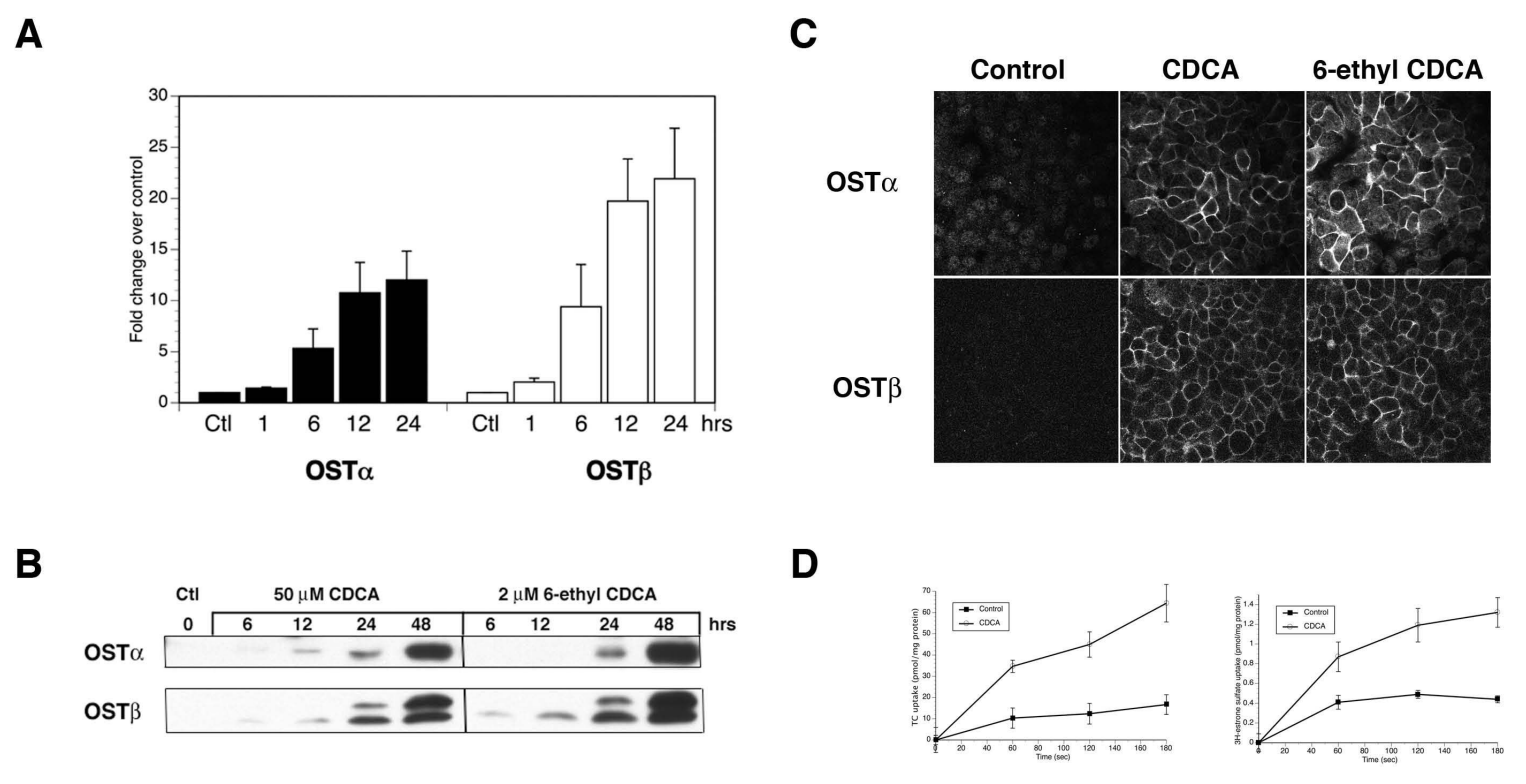
**FXR agonists up-regulate OSTα and OSTβ mRNA and protein in HepG2 cells.** (A) mRNA was isolated from HepG2 cells treated with CDCA for the indicated time periods. Quantitative RT-PCR indicates that treatment with 50 μM CDCA up-regulates mRNA for OSTα (closed bars) and OSTβ (open bars) in a time dependent manner, peaking between 12 and 24 hrs. (B) Lysates from HepG2 cells treated with CDCA for the indicated time periods were combined with Laemmli sample buffer and subjected to SDS-PAGE. Protein expression for both subunits is detectable between 12 and 24 hrs and increases significantly at 48 hrs as demonstrated by Western blotting using the specific polyclonal antibodies described in Methods. (C) Immunofluorescence on cells treated for 48 hrs with vehicle alone, 50 μM CDCA or 2 μM 6-ethyl CDCA demonstrates that both the up-regulated OSTα and OSTβ subunits are correctly expressed on the plasma membrane. (D) Transport studies in HepG2 cells using ^3^H-taurocholate (1 μM) and ^3^H-estrone 3-sulfate (50 nM) indicate that treatment with 50 μM CDCA up-regulates functional activity 3–4 fold over non-treated cells.

Previous studies indicate that human hepatocytes express OSTα-OSTβ on their basolateral membranes [4]. In untreated human hepatoma, HepG2, cells, there was little expression of OSTα-OSTβ mRNA or protein. However, treatment with the FXR agonist, CDCA, up-regulated mRNA levels of both subunits rapidly within one hour and peaked between 12 and 24 hours. mRNA levels increased ~12 fold for OSTα and ~20 fold for OSTβ (Figure 1A). Protein levels for both subunits were not detectable until at least 12 hrs after treatment with 50 μM CDCA or 2 μM 6-ethyl CDCA (Figure 1B). Both subunits were visualized on the plasma membrane at that time, with 70–90% of cells expressing the transporter after 48 hrs (Figure 1C). A time course using immunofluorescence detected no OSTα prior to 12 hrs, although OSTβ could be visualized in a perinuclear localization at earlier time points (data not shown). Finally, uptake of both ^3^H-taurocholate and ^3^H-estrone 3-sulfate was increased 3–4 fold in CDCA treated cells, indicating that OSTα-OSTβ was functional in these cells when expressed on the plasma membrane (Figure 1D). Uptake of taurocholate can also be mediated by other basolateral transporters such as the sodium taurocholate co-transporting protein (NTCP) and the family of organic anion transporter proteins (OATPs). Therefore, we also measured mRNA levels for OATP1A2, OATP1B1, OATP1B3, and NTCP by Q-PCR. While most were unchanged after CDCA treatment, mRNA for OATP1B3 was up-regulated (data not shown), consistent with previous reports [9]. Thus, we cannot rule out that some of the uptake was due to OATP1B3.

**Figure 2:**
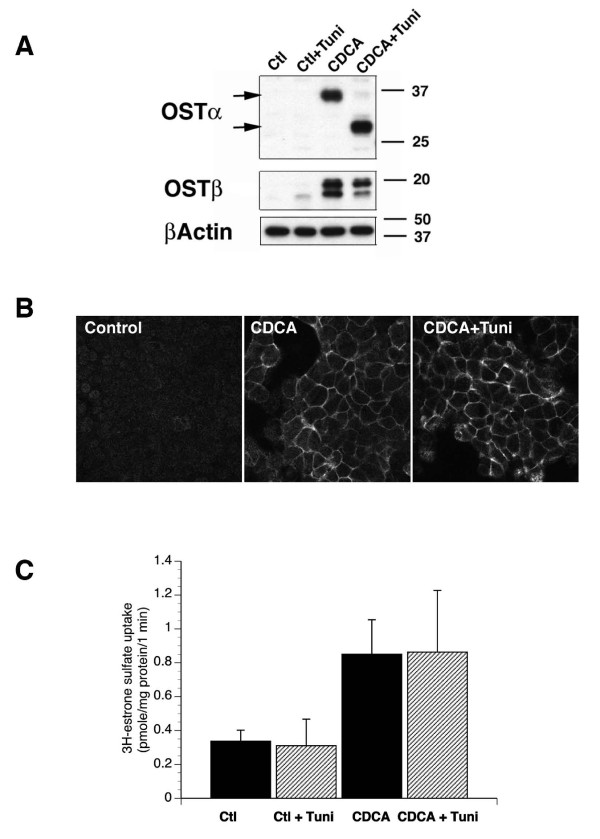
**Tunicamycin prevents glycosylation of OSTα but does not affect its plasma membrane localization or its transport function.** Tunicamycin (1 μg/ml) was added to HepG2 cells 5 hrs after the addition of 50 μM CDCA and incubation continued for a total of 15 hrs. Cells were then extracted with RIPA buffer for PAGE and Western blotting, fixed for immunofluorescence, or subjected to the transport assay as described in Methods. (A) Western blot analysis of cell lysates indicates that the molecular weight of the OSTα subunit was reduced from ~36 kD to ~28–30 kD (see arrows), indicating that this subunit is glycosylated. The molecular weight of the OSTβ subunit was not significantly changed and β-actin was used as a loading control. (B) Immunofluorescence for OSTα shows that tunicamycin treatment did not prevent expression of the alpha subunit on the plasma membrane. (C) Transporter function as assessed by ^3^H-estrone 3-sulfate uptake was also not affected by treatment with tunicamycin. n = 3

Examination of the amino acid sequence of the alpha subunit has indicated that, unlike in the mouse, rat and skate sequences, the human OSTα subunit does not have the traditional N-glycosylation consensus sequence of Asn-X-Ser/Thr [1]. Therefore, it was of interest to determine if the human OSTα protein was glycosylated. Tunicamycin is an antibiotic that has been shown to inhibit N-glycosylation of proteins by blocking the addition of N-acetylglucosamine to dolichol phosphate, the first step in the formation of core oligosaccharide [10]. Treatment of HepG2 cells with tunicamycin (1 μg/ml), 5 hours after the addition of 50 μM CDCA and for a total of 15 hrs, reduced the molecular weight of OSTα from ~36 kD to ~30 kD (Figure 2A). Treatment with the glycosidases, Endo H and PNGase F, confirmed that these proteins were the mature and non-glycosylated forms of OSTα (data not shown). There was no shift in the molecular weight of the OSTβ subunit after tunicamycin treatment. This confirms previous data demonstrating that the only potential N-glycosylation consensus sequence in mouse Ostβ is in a presumably transmembrane domain and, thus, is not utilized [6]. These data indicate that endogenously expressed human OSTα is a glycoprotein. However, despite the lack of N-glycosylation in these treated cells, immunofluorescence demonstrated that the OSTα subunit was still expressed on the plasma membrane at similar levels to untreated cells (Figure 2B). Furthermore, the tunicamycin treated HepG2 cells showed no difference from untreated cells in ^3^H-estrone 3-sulfate uptake, demonstrating that the non-glycosylated OSTα was still capable of associating with OSTβ to form a functional transporter (Figure 2C). In contrast, previous work has suggested that N-glycosylation of Oatps is essential to their functioning[11,12]. The lack of difference in uptake after tunicamycin treatment suggests that little, if any, of the ^3^H-estrone 3-sulfate transport was through OATPs.

### Plasma membrane expression of OSTα requires OSTβ, but not N-glycosylation

**Figure 3:**
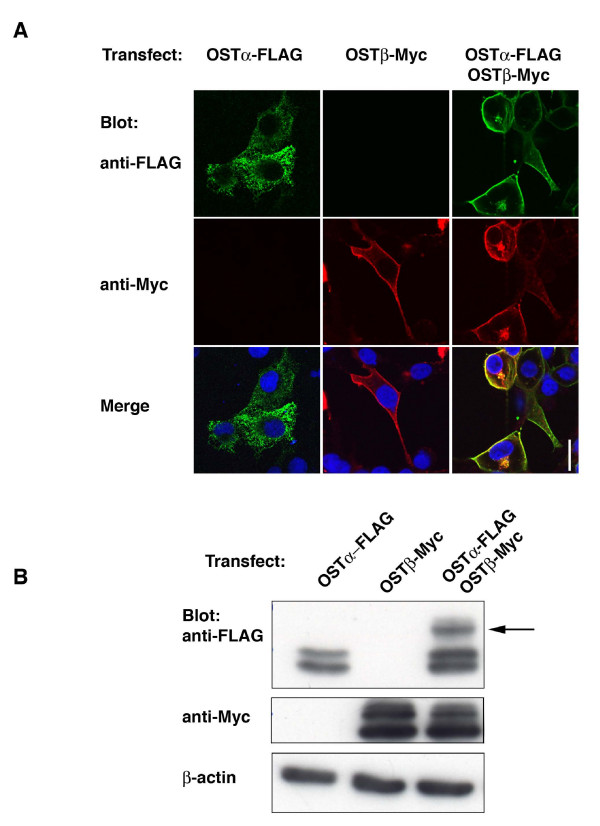
**Membrane expression of OSTα requires the co-expression of OSTβ in COS7 cells.** (A) COS7 cells were transfected with OSTα-FLAG and/or OSTβ-Myc as described in Methods and immunofluorescence was performed to visualize the localization of the individual subunits using mouse anti-FLAG and rabbit anti-Myc antibodies. Transfection of OSTα-FLAG (green) alone indicates that in the absence of OSTβ, OSTα is retained intracellularly (left row). In contrast, transfection of OSTβ (red) alone shows that this subunit can traffick to the plasma membrane without OSTα (middle row). Co-expression of OSTα and OSTβ subunits results in plasma membrane localization of OSTα (right row). Bar= 10 μM. (B) Transfected cells were also lysed with RIPA and lysates subjected to PAGE and Western blotting. When OSTα-FLAG and OSTβ-Myc were co-transfected a higher molecular weight band for OSTα was detected (arrow), suggesting a mature, glycosylated form of OSTα (B).

COS7 cells were transfected with OSTα-FLAG and OSTβ-Myc in order to evaluate the behavior of the individual subunits. When OSTα-FLAG alone was transfected, immunofluorescence revealed only an intracellular signal and never detected protein on the plasma membrane (Figure 3A). However, when both OSTα-FLAG and OSTβ-Myc were transfected together, both proteins were detected on the plasma membrane by immunofluorescence (Figure 3A). Transfection of OSTβ-Myc alone resulted in plasma membrane and intracellular localization (Figure 3A), demonstrating the ability of Ostβ to traffic to the plasma membrane independently of Ostα in an over-expressing system. This has also been reported in transfected MDCK and HEK293 cells [7,8].

**Figure 4:**
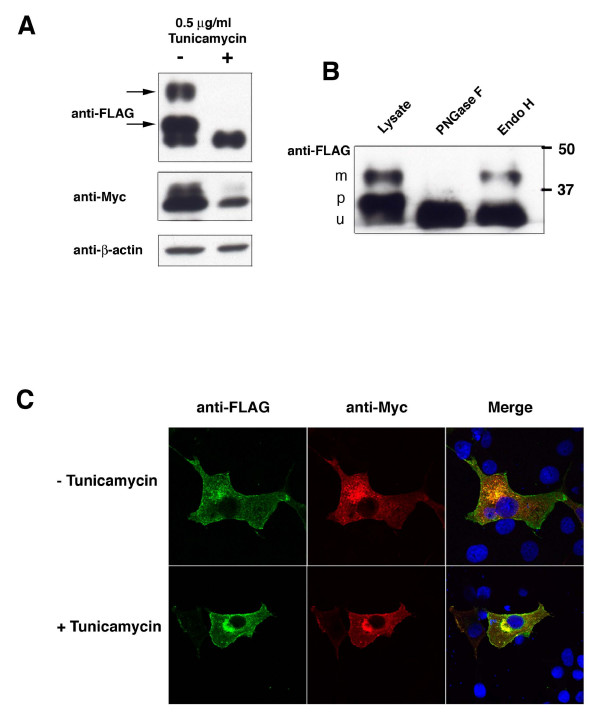
**The mature form of OSTα is glycosylated when co-transfected with OSTβ, but this is not necessary for plasma membrane localization.** COS7 cells were treated with 0.5 μg.ml of tunicamycin or vehicle at the time of transfection and cells were cultured for an additional 48 hrs. (A) Proteins were separated by PAGE, transferred to PVDF membrane, and OST subunits were detected with anti-FLAG and anti-Myc antibodies. The two higher molecular weight bands (arrows) are not detectable after inhibition of glycosylation with tunicamycin. (B) The two higher molecular weight bands (m and p) are sensitive to digestion with PNGase F, but not Endo H, indicating that they contain complex oligosaccharides. m = mature, p = precursor, u = unglycosylated. (C) The lack of glycosylation of OSTα after tunicamycin treatment does not prevent its plasma membrane localization. Anti-FLAG-green, anti-Myc-red

Western blotting revealed that transfection of OSTα-FLAG alone resulted in two bands of approximately 31 and 35 kD (Figure 3B). Co-expression of OSTβ with OSTα resulted in an additional, higher molecular weight species of OSTα of ~40 kD (Figure 3B). These data are similar to that reported for mouse ileum and for HEK293 cells transfected with mouse Ostα and Ostβ [6], and suggest that this band represents a mature, complex glycosylated form of the OSTα subunit. Indeed, when N-glycosylation was inhibited with tunicamycin (0.5 μg/ml, 24 hrs) this 40 kD band disappeared (Figure 4A). In addition, the 35 kD band also disappeared, suggesting that this band represents the core glycosylated or precursor form found in the ER. The 31 kD band was not eliminated by tunicamycin treatment indicating that it is the non-glycosylated OSTα subunit. In contrast to HepG2 cells, tunicamycin treatment of COS7 cells appeared to reduce the expression of a higher molecular weight band of the OSTβ subunit (Figure 4A), although this was not a consistent finding. This subunit is not glycosylated, thus the change may reflect a non-specific effect of the tunicamycin treatment in this experiment.

The glycosylation status of OSTα was further clarified by treatment of cell lysates with the glycosidases, Endo H and PNGase F. These two enzymes can distinguish between N-glycans that only contain the core oligosaccharide that has been added in the ER (Endo H sensitive) and those that have trafficked through the Golgi and have had their carbohydrate chains modified (PNGase F sensitive, Endo H resistant) [13]. Figure 4B shows that the 40 kD band was sensitive to PNGase F, but not Endo H, treatment, indicating that the mature alpha subunit has exited the Golgi. The 35 kD band was sensitive to both Endo H and PNGase F and, thus, represents a glycoprotein that has not trafficked through the Golgi. The 31 kD band remains after both glycosidase treatments, confirming that it represents the non-glycosylated OSTα subunit. These data demonstrate that human OSTβ is required for human OSTα to be processed from the high mannose type N-linked glycan in the ER to complex oligosaccharides in the cis/medial Golgi region. Immunofluorescence of tunicamycin treated COS7 cells transfected with OSTαFLAG and OSTβ-Myc showed that the lack of glycosylation did not prevent OSTα trafficking to the plasma membrane, confirming data seen in HepG2 cells (Figure 4C). Thus, the interaction of the beta subunit with the alpha subunit in the ER and the subsequent trafficking through the Golgi does not require that OSTα be glycosylated.

### Immunoprecipitation demonstrates that immature forms of OSTα and OSTβ associate

**Figure 5:**
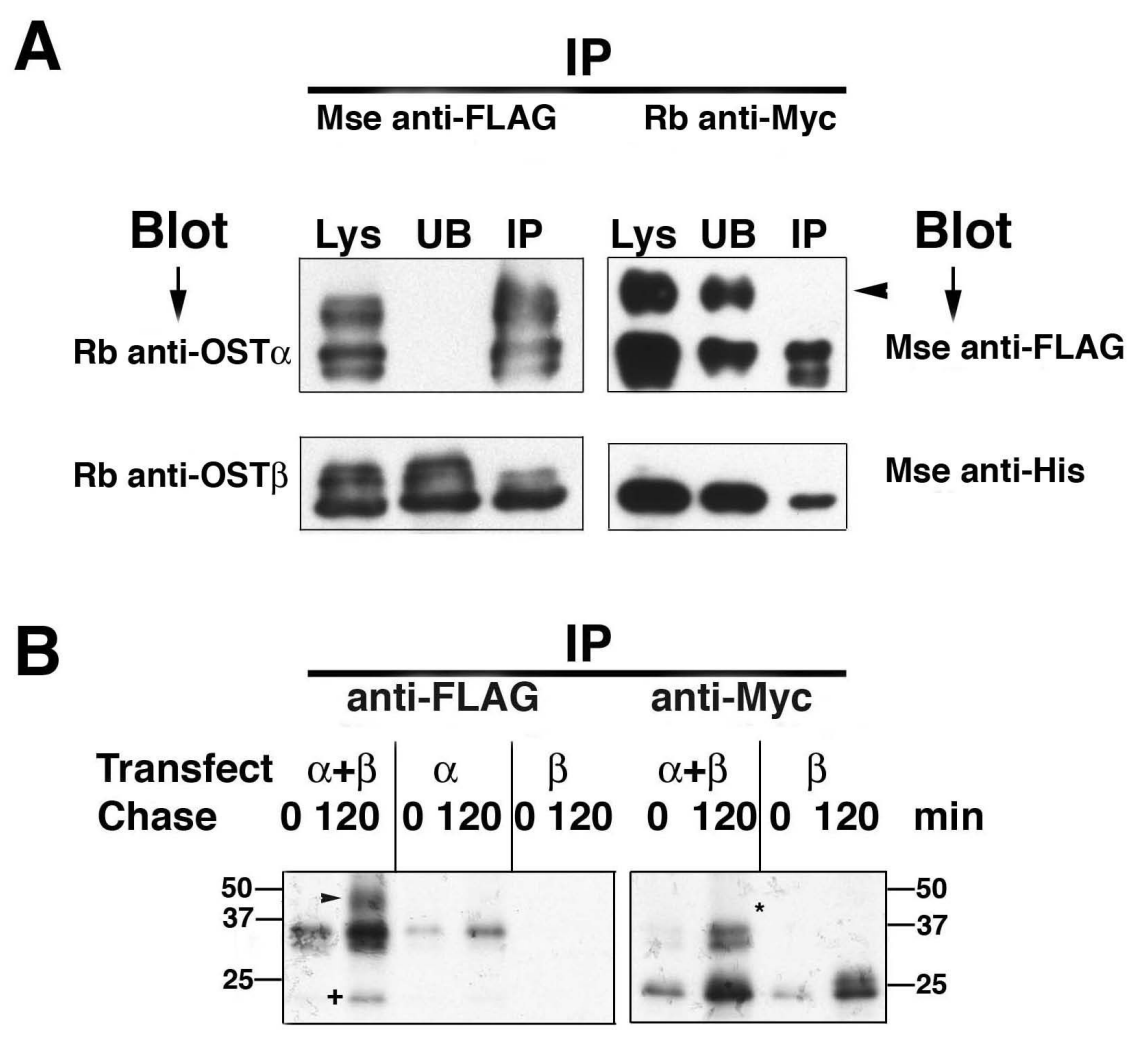
**Immunoprecipitation experiments indicate that the intracellular, immature OSTα and OSTβ are physically associated.** (A) Lysates from COS7 cells transfected with OSTα-FLAG and OSTβ-Myc for 48 hrs were subjected to immunoprecipitation using mouse anti-FLAG agarose beads (left two panels) or rabbit anti-Myc and proteinA/G beads (right two panels). Following PAGE and transfer to PVDF membrane the blots were probed with rabbit antibodies to OSTα (top left panel) and OSTβ (bottom left panel) or mouse antibodies to FLAG (top right panel) and His (bottom right panel), respectively. As noted in the Methods, OSTβ-Myc also contains a His tag. Anti-FLAG agarose efficiently removes all OSTα-FLAG from the lysate (Lys), however, it only co-precipitates a portion of OSTβ(left two panels). Although anti-Myc was rather inefficient in removal of OSTβ-Myc from the lysate, it was capable of co-precipitating the immature forms of OSTα(right two panels). The mature, glycosylated OSTα (arrowhead) was never detected in the precipitate. (B) COS7 cells transfected with OSTα alone, OSTβ alone, or both subunits were subjected to metabolic labeling and immunoprecipitation. Twenty-four hours after transfection cells were pulsed for 15 min with ^35^S-Trans label. Cells were either lysed immediately (0 chase) or chased for 2 hrs (120 chase). Immunoprecipitation was carried out using mouse anti-FLAG agarose beads (left panel) or rabbit anti-Myc and proteinA/G beads (right panel). The left panel shows that the higher molecular weight band (glycosylated form of OSTα) is only present in cells transfected with both subunits, and only after the 2 hr chase (arrowhead). A time course indicated that it was detectable after 30 min of chase (data not shown). The ~20 kD OSTβ subunit is co-precipitated from these cells (+). In contrast, immunoprecipitation of OSTβ with anti-Myc antibody resulted in co-precipitation of only the immature form of OSTα (right panel). The asterick (*) indicates the position where the mature form would have appeared.

In order to gain further insight into the interaction of the two subunits, immunoprecipitation was conducted using antibodies to the tags associated with both the alpha and beta subunits. In COS7 cells, when OSTαFLAG was immunoprecipitated with anti-FLAG agarose beads, the precipitate also contained OSTβ (Figure 5A). Although all the OSTα was efficiently precipitated, only a fraction of the OSTβ was found in the precipitate. When OSTβ was immunoprecipitated with anti-Myc antibody, only the two lower molecular weight forms of OSTα were found in the precipitate (Figure 5A). Despite repeated attempts using more protein and over-exposure of the blots (data not shown) the mature, complex glycosylated form of OSTα was never seen. Metabolic labeling of transfected COS7 cells demonstrated that the 40 kD form of OSTα appeared only after co-transfection of the alpha and beta subunits and after a time lag of > 15 min (Figure 5B; earlier time points not shown). Furthermore, anti-FLAG precipitated all forms of OSTα as well as OSTβ, but anti-Myc co-precipitated only OSTβ and the immature forms of OSTα (31 and 35 kD bands), confirming the previous data. This suggested that the physical association of the two subunits may be necessary for the transporter to be processed and trafficked through the intracellular compartments, but possibly not necessary once OSTα reaches the plasma membrane.

**Figure 6:**
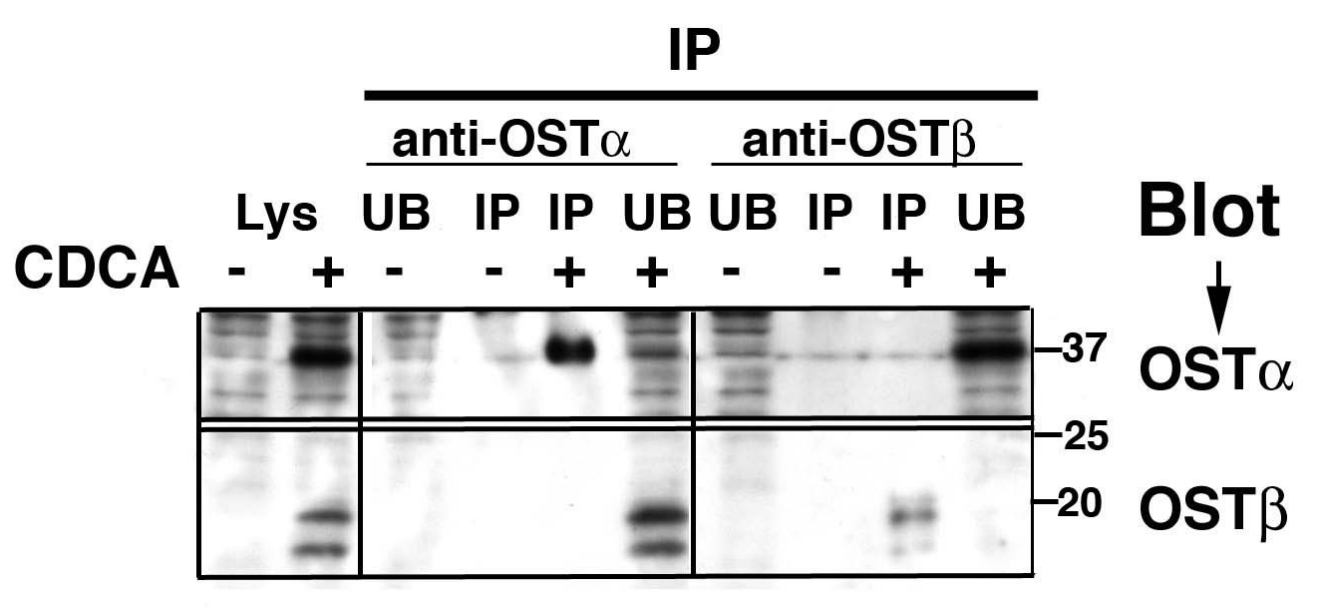
**Immunoprecipitation of endogenous protein in HepG2 cells further support the data that co-precipitation does not occur between the mature forms of OSTα and OSTβ.** HepG2 cells were treated for 48 hrs with CDCA and then lysed with 1% Triton X-100 buffer as described in Methods. Cells extracts were subjected to immunoprecipitation using rabbit anti-OSTα (middle panel) or rabbit anti-OSTβ (right panel) and the immunoprecipitates (IP) and the fraction not bound to the Protein A/G beads (UB) were separated by SDS-PAGE. As a positive control, the cell lysate (Lys) was also subjected to SDS-PAGE (left panel). Western blot was then performed using the same polyclonal antibodies and a Native IgG kit from Pierce. In the case of HepG2 cells, only the mature form of OSTα is detected in the lysate. Co-immunoprecipitation of OSTα and OSTβ does not occur, although the proteins are clearly detectable in the unbound fractions. Lys = lysate; UB = unbound fraction; IP = immunoprecipitated, bead fraction

To test whether this could be an artifact of transfection of exogenous DNA, we also conducted immunoprecipitation of endogenous protein in HepG2 cells. In this case it should be noted that after CDCA treatment we see expression only of the mature form of OSTα (Figure 2A), suggesting that the immature form(s) move very rapidly through the Golgi and are of too low abundance to detect. Immunoprecipitation using the polyclonal anti-OSTα and OSTβ antibodies does not demonstrate co-precipitation of the two subunits (Figure 6A). This is consistent with the lack of association between the mature, plasma membrane subunits.

## Discussion

The importance of the novel heteromeric, basolateral transporter, Ostα-Ostβ, in enterohepatic circulation of bile acids and the homeostasis of bile acid synthesis has recently been confirmed [5]. Although it is clear that function of this facilitated transporter requires expression of both subunits, it is not known whether functional activity depends upon (1) the acquisition of N-glycosylation of the alpha subunit, (2) the beta subunit for its ability to release the alpha subunit from an ER retention signal, or (3) the physical interaction of the two proteins at the plasma membrane. The data provided here indicate that glycosylation of OSTα is not necessary for transporter localization or function. Furthermore, it shows that the physical interaction of the two subunits may be transient, suggesting that association at the plasma membrane may not be necessary for transporter function.

Glycosylation of a protein is one of the major biosynthetic functions of the ER and is a common post-translational modification of membrane proteins. Although the addition of the "core" oligosaccharide occurs in the ER, further extensive processing or trimming occurs in the Golgi and results in what is commonly referred to as the complex or mature glycoprotein [13]. N-glycosylation is found usually in the sequences Asn-X-Ser or Asn-X-Thr, where X is any amino acid [13,14]. Although this consensus motif is found in the N-terminus of the alpha subunit in the mouse, rat and skate, it is not present in the human OSTα [1]. Instead, the sole asparagine residue in an extracellular site is in the sequence Asn^25^-X-Gly in the N-terminus. We have shown in this study that, despite the lack of traditional consensus sequence, human OSTα is expressed on the cell surface as a glycoprotein. Similar to previous reports [3,4,6,7,15] our data indicate that endogenous alpha subunit migrates in SDS-PAGE as a single band and precursor forms are not detected. This suggests that in the presence of the beta subunit the glycoprotein is efficiently trafficked through the Golgi. It is only in the over-expressing transfected cells that the multiple forms of the alpha subunit are seen (Figure 3, 4 and 5 this manuscript; [3,6].

The necessity for glycosylation of proteins has been studied for many years and is largely believed to be important in proper folding and stabilization of newly synthesized proteins and in affecting the charge and solubility of the protein [16,17]. The critical nature of this folding is highlighted by the finding that detection of misfolded glycoproteins in the ER can result in ER-associated degradation (ERAD) [18,19]. Our data indicate that the lack of oligosaccharide chain on the alpha subunit does not designate the polypeptide for ERAD. Instead, after tunicamycin inhibition of glycosylation, the transporter was still trafficked properly to the plasma membrane where it was fully functional, indicating that interaction between the alpha and beta subunits is not compromised by the lack of oligosaccharide. Perhaps because the alpha subunit of the organic solute transporter has only one asparagine residue in an extracellular domain[1], the affect of the absence of the carbohydrate on folding is not critical. Tunicamycin treatment has been used to study glycosylation of other hepatocyte proteins. The absence of oligosaccharide did not affect the secretion of transferrin or very low density lipoprotein[20], but did interfere with the ability of the apical membrane protein, Mrp2, to be trafficked to the plasma membrane in rat hepatocytes [21]. And recently the N-linked carbohydrates have been described for the hepatocyte basolateral membrane protein oatp1a1 and found to be important in the protein's localization and function [12]. In HepG2 cells it has been reported that five of eight glycoproteins studied did not require glycosylation for their trafficking [22]. Mochizuki et al have shown that rat Bsep requires at least two of its four N-linked glycans for proper protein stability, intracellular trafficking and functional activity [23].

Interestingly, we (Figure 5B) and others [5,7] have shown that the absence of one of the subunits leads to degradation of the other subunit. Thus, it is the presence and interaction of the two subunits that are critical to the stability of the heteromeric, intact transporter, and not the glycosylation of the alpha subunit. Protein-protein interactions in the ER are known to be critical for many different processes, including trafficking and function of multimeric membrane proteins. The presence of fully functional oligomeric complexes at the plasma membrane can involve specific ER retention/retrieval motifs[24,25], anterograde ER export signals [26,27], interaction with scaffold protein [28-31], and phosphoylation [29,32]. The necessity for interaction between OSTα and OSTβ subunits in the ER suggests that physical association of the two proteins may mask a retention/retrieval motif or, alternatively, may reveal a forward trafficking motif. The RXR motif is one such retention/retrieval sequence and it is interesting that both the alpha and beta subunits contain an RXR-like motif in their C-terminal sequence. It remains to be determined whether this sequence is important in the localization of the organic solute transporter.

Our immunoprecipitation data confirm that the OSTα and OSTβ interaction is essential early in the biosynthetic process, but suggest that it may not be necessary later once the major protein gets to the plasma membrane. Because the only way to get OSTα to the plasma membrane is to co-express the beta subunit, it is impossible to determine if the alpha subunit actually requires the beta subunit for its functional activity. However, the lack of co-precipitation between the mature form of OSTα and the OSTβ subunit suggests that this may not be the case. When Li and colleagues [7] performed similar immunoprecipitations in HEK293 cells transfected with mouse Ostα and Ostβ constructs, they also saw only a single band after precipitation with anti-Myc. However, they indicate that it is the mature form of the protein. Given that all data point to the interaction of the subunits in the ER, one would also expect to see the immature form precipitated. Similarly, in mouse ileum Li et al show only one band for Ostα on Western blots and this protein is co-precipitated by an antibody to Ostβ [7]. Although the explanation for these differences in immunoprecipitation is still unclear, we cannot discount that it is due to species variability or species-specific antibody affinity.

The possible transient nature of the subunit interaction also appears to be in conflict with immunofluorescent studies which suggest co-localization of the subunits at the plasma membrane in transfected cells (Figure 2 and [8]. However, the finding of a yellow color indicating co-localization may be due to the close proximity of the two subunits, not the actual association. Optical microscopes are unable to resolve two items that are closer together than 200 nm. Also, we cannot discount the possibility that, similar to tunicamycin treated cells, some "immature" protein might be expressed on the plasma membrane, and, thus, be detected by the primary antibodies. Bimolecular fluorescence complementation has also been used to study the interaction of the two subunits in HEK293 cells transfected with mouse Ostα and Ostβ [7]. These studies clearly show that complementation occurs between Ostα and Ostβ and results in plasma membrane localization. However, the possibility that the interaction might be transient cannot be assessed because, once the complementation reaction occurs, it is irreversible.

## Conclusion

In conclusion, this study demonstrates that, although human OSTα is a glycoprotein, the carbohydrate chains are not necessary for interaction with OSTβ or subsequent exit from the ER. Furthermore, plasma membrane localization and functional activity of the organic solute transporter does not depend upon N-glycosylation. Interaction between the two subunits occurs early in the biosynthetic pathway, but may not be necessary at the plasma membrane.

## Abbreviations

CDCA: chenodeoxycholate; Endo H: endoglycosidase H; ER: endoplasmic reticulum; FXR: farnesoid × receptor; OST/Ost: organic solute transporter; PNGase F: peptide:N-glycosidase F

## Authors' contributions

CJS designed and carried out all experiments, except Q-PCR, and was responsible for the writing of the manuscript. SX designed and constructed the OSTα-FLAG and OSTβ-Myc/His. AM carried out all Q-PCR. PL performed the Endo H and PNGase F assays. JLB participated in the design of the study and in all discussion of the data. All authors read and approved the final manuscript.
